# DNA and mRNA Vaccines for Chronic Viral Infections and Cancer: Rationale, Mechanisms, and Progress

**DOI:** 10.3390/cancers14235874

**Published:** 2022-11-29

**Authors:** Margaret A. Liu

**Affiliations:** 1 ProTherImmune, 3656 Happy Valley Road, Lafayette, CA 94549, USA; liu@protherimmune.com; Tel.: +1-925-299-2959; 2 Department of Medicine at Solna, Karolinska Institutet, 17177 Stockholm, Sweden

**Keywords:** DNA vaccines, mRNA vaccines, cancer, chronic viral infections, immunotherapy, immunotherapeutic vaccines

## Abstract

**Simple Summary:**

This review describes the rationale supporting the use of DNA and mRNA vaccines as immunotherapeutic vaccines for treating chronic viral infections and cancer. It specifically focuses on the immunological challenges for generating potent therapeutic responses when an infection or tumor are already established, then describes the immunologic capabilities of DNA and mRNA vaccines that may enable them to be potent enough for curing the infection or treating the cancer.

**Abstract:**

Interest in the capabilities of nucleic acid vaccines, (DNA and mRNA vaccines) for both prophylactic and therapeutic uses have greatly increased following the successful deployment of two mRNA and, on a more limited scale, one DNA vaccine for COVID-19. In addition to targeting other pathogens for prophylactic vaccines, efforts are also being made towards using them for therapies for chronic infections and cancer. An examination of past and current successes for such therapies using other technologies with an emphasis on the immunological mechanisms will be provided followed by an assessment of the relevant characteristics of DNA and mRNA vaccines to predict their utility for therapies for chronic viral infections and cancer. Efforts and progress for these targets will be described.

## 1. Introduction

Because certain cancers and chronic viral infections may in some regards be thought of as a failure of the immune system to adequately respond to tumor and viral antigens or due to escape of the tumor from the immune responses [1], vaccines or immunotherapeutic approaches are being developed as antigen-specific immune therapies for chronic viral infections and cancer, whether the cancer is caused by a virus or not. Nucleic acid vaccines, both DNA and mRNA vaccines have characteristics that make them potentially well-suited for these therapeutic applications. This manuscript will focus on therapeutic vaccines for both chronic infections and cancer in part because up to one-fifth of cancers are thought to be due to viruses [2]. Table 1 illustrates the rationale for considering both chronic infections and cancer by providing examples of pathogens that cause both infectious diseases and malignancies.

Certain unique characteristics of DNA and mRNA vaccines used for prophylactic vaccines make them a promising technology for therapeutic vaccines. The key relevant attributes and advantages compared to other technologies as well as the immunologic issues for immunotherapies will be discussed. These include the rationale for why immune responses may work as therapies, the challenges that are specific to chronic diseases and the tumor environment, the specific types of immunity induced by nucleic acid vaccines, and the potential advantages for antigen design and manufacture that nucleic acid vaccines offer.

## 2. Immunologic Issues

Immunotherapeutic vaccines need to consider both what antigens are appropriate and the immunological milieu. These vaccines need to succeed in activating immune responses to deal with an immunological environment where the antigen has already been present for some time yet virus has already survived despite the immune responses during acute infection, or where a cancer has already arisen despite anti-tumor responses that developed when the cell underwent transformation from a normal cell to a malignant one. For some pathogen-induced cancers, the pathogen is directly responsible for transforming cells: HPV E6 and E7 are oncogenic by various mechanisms [3] and likewise certain EBV proteins play a role in EBV-related malignancies [4]. For other pathogens the interaction is more complex and chronic inflammation plays a role (e.g., HCV). Therefore, the approaches for therapeutic vaccines may need to differ both in terms of the antigens they target and the types of immunity generated compared to a prophylactic vaccine against an oncogenic virus for example.

### 2.1. Considerations for Antigens

Reasons that different antigens may need to be targeted include the fact that, for chronic viral infections, a prophylactic vaccine might target a viral surface protein, whereas a therapeutic vaccine might be more effective by targeting a protein that plays a role in the cellular transformation or that is highly expressed after infection. Licensed HPV prophylactic vaccines target L1, the major capsid protein, whereas E6 and E7 are prime targets for therapeutic vaccines under development because of their role in the transformation of infected cells, leading to cancer as noted above [3]. The hepatitis B prophylactic vaccine utilizes the Hepatitis B surface antigen, yet following hepatitis B infection, clearance of the virus either after acute or chronic infection correlates with both CD4+ and CD8+ T cells directed against core and polymerase proteins in addition to surface proteins [5], suggesting that using only the surface antigen might be less than optimally effective for a therapeutic hepatitis vaccine.

### 2.2. Challenges and Types of Immune Responses

In addition to possibly targeting different antigens, a therapeutic vaccine may need to provoke either stronger or additional/different immune responses than for a prophylactic vaccine and overcome the particular immunological milieu of chronic infection and tumors. In the cases of chronic viral infection, immunotherapeutic vaccines will be given to an individual who already has viral antigens present, perhaps even at high levels (which may contribute to immune exhaustion) [6], so simply providing the same antigens as a vaccine would only generate effective immune responses if the antigens are delivered in such a manner or with immune modulators that would enable potent immune responses in the face of such immune dysfunction. Cellular dysfunction in chronic hepatitis B infection [6,7,8], includes T cell exhaustion with decreased antigen-specific cytokine production, and even T cell apoptosis caused by the continued/repetitive antigen stimulation from the persistent presence of the viral antigens. B cells likewise may not be fully functional and may be depleted. This contrasts with the situation of a prophylactic vaccine where the viral antigen is novel to the vaccinee, and where antigen-specific immune dysfunction would not have been already induced by the virus. Adjuvants, immune stimulators (such as cytokines), and even immune modulators such as checkpoint inhibitors are being evaluated for their ability to stimulate effective immune responses in the setting of chronic infection with its immune escape and exhaustion.

Given these challenges, why is there hope that therapeutic vaccines can be made against chronic viral infections and cancer, and what types of immune responses are needed? Will adaptive immunity, i.e., antigen-specific B and/or T cell responses be sufficient, or is there a role or need for innate immunity? What do nucleic acid vaccines offer as technologies for successful therapeutic vaccines? To answer these questions, we shall first examine the rationale for therapeutic vaccines.

## 3. Rationale for Immunotherapeutic Vaccines for Chronic Viral Infections and Cancer

As seen in Table 2, a number of clinical observations and successful therapies underscore the ability of immunological approaches to suppress if not clear chronic viral infections and to treat cancer. These include, in addition to the obvious success with which the immune system eliminates a number of acute viral infections, the concept of immunosurveillance (dating back to the early 20th century) a term describing the immune system’s ability to destroy certain tumor cells that arise [1]. It is thus thought that only in certain situations do tumors escape the immune responses such that the individual develops clinical cancer [9]. The role of the immune system in normally suppressing tumors that arise is further evidenced by the tumors that arise in immunocompromised states such as in HIV infection, where specific cancers helped to discover (Kaposi’s sarcoma and Non-Hodgkin’s lymphoma) and define (invasive cervical carcinoma) the clinical syndrome of AIDS [10]. Furthermore, as HIV infection directly compromises the T cell compartment by infecting and killing CD4+ T cells, the increased incidence of other cancers (Hodgkin’s lymphoma, anal cancer, lung cancer) was found to inversely correlate with the declining CD4+ T cell counts [10]. Additional evidence for the ability of a normal immune system to suppress cancer development was provided by other immunosuppressed states, such as after organ transplantation, when patients who are on immunosuppressive regimens to decrease the rejection of the transplanted organs had an increase in a variety of malignancies, with an additional aggressiveness of the cancers (rate of tumor growth and spread) [11].

The rationale for immunotherapy generally, and for immunotherapeutic vaccines specifically, arose from a variety of observations and approaches. These ranged from non-specific immunostimulation, administration of cytokines, and the use of checkpoint inhibitors, to approaches such as monoclonal antibodies, bispecific antibodies, and CAR-T cells.

These approaches will be briefly described because they lay the groundwork for the rationale and design of nucleic acid therapeutic vaccines.

### 3.1. Non-Specific Immunostimulation

Towards the end of the 19th century, a physician named William Coley observed a patient with sarcoma that kept recurring despite repeat surgery, whose wound became infected with erysipelas (*Streptococcus pyogenes*). The tumor was noted to shrink each time the patient had a high fever, such that he had a complete remission of the tumor. Coley thus began to test ways to reproduce this first by infecting patients, (which had a variety of problems including that this was the pre-antibiotic era, so some patients died from the infection), and then by the use of a “vaccine” (even though it was not actually a vaccine) composed of two killed bacteria: *S pyogenes* and *Serratia marcescens*, which became known as “Coley’s toxin” [12,13]. While for various reasons, this approach fell out of favor, the concept of finding a role for non-specific inflammation, as well as, or combined with, specific adaptive immunity has increased in recent years as it has become clear that the cancer or chronic viral environment may need more than simple provision of a tumor or viral antigen.

BCG or the Bacillus Calmette Guerin, the mycobacterium that was developed into a prophylactic vaccine for tuberculosis has also been licensed as a therapy to prevent progression or recurrence of bladder cell cancer even though, again it is not actually a vaccine for the tumor. The exact mechanisms are not known but are thought to include induction of cytokines and non-specific effects of inflammation as well as tumor-specific immune responses [14,15] that arise from the inflammation.

Neither of these approaches is a vaccine, but rather they demonstrate that the bacteria induce inflammation and innate immune responses that result in cancer cell death even though the bacteria themselves do not deliver tumor antigens to generate tumor-specific immunity (such as tumor-specific T cells or antibodies). (Any tumor-specific immune responses that arise after bladder BCG administration arise due to the immune responses against tumor cells killed by the inflammation from the BCG.)

### 3.2. Immune-Mediated Cancer Therapies

The demonstration of the ability of antigen-specific immune responses to have a therapeutic impact on existing cancers has included the demonstration of the effectiveness of both antibodies and T cell responses. We may be able to glean from these approaches an understanding of what may be useful or needed to be designed into nucleic acid vaccines in order for them to be successful as immunotherapies.

#### 3.2.1. Cytokines

IFNα was approved by the US FDA for treatment of cancer in 1986 [16] for the treatment of chronic Hepatitis B and C as well as various cancers. Its usage and activity are of further relevance for this discussion of nucleic acid vaccines because DNA, RNA and nucleotides stimulate many types of cells to produce both IFNα and IFNβ. Recombinant Il-2 was approved for the treatment of metastatic kidney cancer in 1992 and for metastatic melanoma in 1998. IL2 stimulates T cell responses, thus also supporting the concept of other T cell therapeutic approaches to cancer, such as CAR-T cell therapies and checkpoint inhibitors [17].

#### 3.2.2. Monoclonal Antibodies Including Bispecific (Heterobifunctional) Antibodies

Various monoclonal antibodies (MAbs) have been approved for the treatment of cancer. It is worthwhile remembering that while the concept of using monoclonal antibodies as “silver bullets” against cancer was a hoped-for use soon after the development of hybridoma technology to make such antibodies in 1975 [18], it took over two decades of work to transform the concept into reality with the 1997 licensure of rituximab for lymphoma. This was due to the needs of finding the optimal tumor targets, necessary modifications of the antibodies themselves, and of course determining the clinical protocols for administration. MAbs are of course different from a traditional vaccine, where the focus is on presenting an antigen to the immune system with the subsequent development of a new immune response, since MAbs themselves can be designed to already = bind to tumor antigens. Two different types of MAbs (or a different ligand plus monoclonal antibody) can also be linked together, with one MAb targeting a molecule on the surface of a tumor cell, and the other MAb targeting a T cell, to specifically bring T cells to the tumor cell to kill the latter independent of the specificity of the T cell receptor. This technology, pioneered by the author initially termed “heteroantibody duplexes” in 1985 [19,20] when first published, is now known as “bispecific antibodies.”

#### 3.2.3. T Cell Specific Mechanisms

Both IL-2 and bispecific antibodies that engage and activate T cells underscore the role of T cells for targeting cancers because they demonstrate that T cells can be either directed to specific antigens on tumors, or can be activated or re-activated despite what may be an immunosuppressive milieu or specific down-regulation of T cells against tumors. Immunostimulatory cytokines (see above) initially demonstrated the possibility; the development of CAR-T cells, (Chimeric Antigen Receptor T cells in which a patient’s own T cells are transfected to express a T cell receptor specific for the tumor cell are grown in vitro then re-infused into the patient), demonstrated the efficacy of T cells to kill tumor cells. These clinically-licensed types of antigen-specific immunotherapy are listed in Table 3.

As noted above, T cell dysfunction occurs in chronic hepatitis B infection. Tumors can also inhibit immune responses via mechanisms specific to T cells resulting in T cell exhaustion. The molecules CTLA4 (Cytotoxic T Lymphocyte Antigen 4) and PD1 (Programmed Cell Death Protein 1) found on T cells, are so-called checkpoint molecules that, when certain ligands on other cells bind to these molecules, the T cell response is negatively regulated [21]. The result is that T cells become less capable of killing the tumor cells [22]. Antibodies that inhibit the binding of these molecules and their ligands, are termed “checkpoint inhibitors (reviewed in [23]), and have been used to treat a variety of cancers, demonstrating the ability of T cells’ effectiveness against cancer when their activity can be restored. These clinically approved treatments underscore the potential for therapeutic vaccines if they can overcome the tumor-mediated immune inhibitions. The also emphasize the importance of finding mechanisms to activate the immune responses in a situation where the viral infection or cancer has altered and/or suppressed immune responses. The multitude of mechanisms whereby immune responses have become ineffective against ongoing viral infection and tumors include both down-regulation of tumor cell antigens as well as local and systemic impairment of immune responses [24].

## 4. Characteristics of DNA and mRNA Vaccines Relevant to Immunotherapeutic Vaccines

DNA vaccines are simply bacterial plasmids of DNA, generally supercoiled, using a promoter functional in mammalian cells, encoding the gene of interest, in this case, a viral protein or tumor antigen. DNA vaccines have been tested in various formulations, but the licensed veterinary DNA vaccines and the SARS-CoV-2 DNA vaccine authorized in India are simply delivered in a buffered solution, with no special formulation. mRNA vaccines, which rose to prominence due to their rapid development and significant efficacy for SARS-CoV-2 prophylaxis, are comprised of mRNA synthesized from modified nucleosides having less innate immune-stimulatory activity, with the in vitro-transcribed mRNA formulated in lipid nanoparticles [25].

The key characteristics that are appealing are: (1) the speed of construction; (2) the facile ability to personalize the construct to the antigens of a patient’s own tumor; (3) the intrinsic immunogenicity of the nucleic acid vaccines, since nucleic acid vaccines are not simple inert gene delivery systems, but also activate specific pathways of the immune system independent of the antigen encoded by the gene; (4) the ease with which such vaccines can be combined with other immunomodulators, whether entities such as check-point inhibitors, or cytokines, themselves potentially delivered by the DNA or mRNA; (5) the ease of manufacture. The rapidity with which the vaccines can be made for a new pathogen such as SARS-CoV-2 and the fact that the manufacturing process does not need to be re-developed for each new vaccine, regardless of the clinical target provide a great advantage over other technologies for certain applications. While both DNA and mRNA can also be used to transfect cells, such as Dendritic cells to then be administered to patients for the induction of immune responses, this paper deals only with the direct administration of a nucleic acid vaccine to the patient. Table 4 lists the DNA and mRNA vaccine licensures or authorizations. Note that the Equine West Nile Virus (WNV) vaccine and Dog Melanoma immunotherapeutic vaccine are no longer in use.

An important attribute of both DNA and mRNA vaccines has been their overall safety in human clinical trials and usage. Concerns about theoretical integration for DNA vaccines were alleviated by the demonstration that integration did not occur using highly sensitive pre-clinical assays in mice [26,27] and then in the safety of many human clinical trials [28,29]. The mRNA vaccines have been administered in over a billion individuals [30], with limited adverse events [31,32].

## 5. Designing Immunotherapeutic Vaccines for Chronic Viral Infections and Cancer

Over the past few decades, many efforts have been made to design vaccines that targeted either specific viral or cancer antigens. Challenges included (1) determining the optimal antigens, (2) discovering ways to circumvent the down-regulation of immune responses or tolerance from various mechanisms, (3) enabling penetration of immune responses to the tumor, particularly large ones with poor vascularity.

### 5.1. Antigens

As noted above, for chronic viral infections, optimal target antigens may include proteins different than those used for a prophylactic vaccine. The most commonly targeted HPV proteins for cancer vaccines in development (including DNA vaccines) are E6 and E7 for HPV whose function enables cellular transformation (reviewed in [33]). Other HPV proteins such as E1 and E2 may also be useful to target [34]. Selection might be based on the proteins targeted by immune responses that successfully clear acute or even, rarely, chronic infection, such as polymerase and core proteins of Hepatitis B. For tumors without an infectious etiology, considerations include selecting an antigen that is unique to the tumor or greatly over-expressed by the tumor compared to normal tissue. This might be a protein that played a role in the transformation of a normal cell to a tumor cell, or a protein that plays a role in the metastasis of the tumor.

A key issue for both viral and tumor antigens is that epitopes of antigens may differ not just between tumor types, or viral etiologies, but between individual patients. Importantly, the T cell epitopes that are derived from tumors are specific for the patient’s MHC haplotype, since those epitopes must bind to either MHC Class I or II molecules for activating CD8+ and CD4+ T Cells, respectively.

Successful CAR-T cell therapies are licensed for treating certain blood cancers, targeting the CD-19 or B Cell Maturation Antigen (BCMA), made for each individual patient from monocytes taken from the patient [35]. However, if one wanted to target the idiotype of the tumor (or any other unique tumor marker) via a therapeutic *vaccine* it would be more feasible with nucleic acid vaccine technology than with CAR-T cell technology, because of the rapidity of their construction and the essentially generic manufacturing process of DNA or mRNA compared to the complexities of growing an individual’s cells from a patient then re-infusing them, as is done for CAR-T cells. The earliest efforts for personalized cancer therapy efforts for nucleic acid vaccines were done pre-clinically and clinically using DNA vaccines for B cell lymphomas in the 1990s because the idiotype of the tumor could be sequenced and rapidly made into a DNA plasmid, which took a matter of 1–4 weeks compared to the 3–4 months needed to make a hybridoma of the anti-idiotype, then to produce enough antibodies and cross-link the antibodies with a carrier [36,37,38,39].

For mRNA, the concept of directly administered individualized therapeutic cancer vaccines has included two general approaches: (1) determining the tumor’s genetic sequence and comparing it to the patient’s profile from healthy cells, then making mRNA coding for mutant proteins predicted to be good tumor antigen targets; (2) utilizing pre-made libraries of mRNA encoding tumor antigens whereby different combinations of mRNAs could be used together depending on a patient’s unique tumor antigens determined by sequencing the genetic material of the tumor. The custom-made vaccines can be made in 1–2 months, more rapidly than using traditional vaccine technologies. However, since starting immunotherapy as quickly as possible is desirable before further tumor growth, even faster approaches are also being developed such as the pre-made libraries of different tumor antigens that can then be combined for any given individual. It should be understood that although the vaccine constructs can be quickly made, as was seen with both the original COVID-19 vaccines and the more recent variant boosters, the clinical development pathway for cancer vaccines will take longer for various reasons including the human trials needed to show clinical benefit. In a recent interview, the founder of BioNTech, Uğur Şahin, predicted that BioNTech’s cancer vaccines, which the company has been working on since before the COVID-19 pandemic, could be available “before 2030.” [40].

As noted above, simply providing antigens may not be enough for cancer vaccines due to many factors, including the potential poor intrinsic immunogenicity of the antigens as well as the immunological milieu of the tumor which may inhibit immune responses. Approaches combining with other modalities will be described below.

### 5.2. Potential Contributions and Issues of Innate Stimulation by Nucleic Acid Vaccines

The observations that tumors as well as their surrounding stroma can inhibit immune responses have led to efforts to not simply increase the immunogenicity of immunotherapeutic vaccines by traditional adjuvants, but also to focus on harnessing the inherent immune activation capabilities of the nucleic acid vaccines themselves. Both DNA and mRNA function as more than simple gene delivery systems because they both have capabilities for stimulating innate immune responses that could contribute to the development of the specific immunity against the encoded antigen(s). One reason that it took longer for mRNA to be more broadly developed for vaccines was actually the need to decrease the inflammatory nature of the mRNA itself.

In vitro transcribed mRNA has a variety of immune activities, such that the discovery that using modified nucleosides decreased the toxicity of mRNA was a key step (along with the development of Lipid NanoParticles (LNPs) for their formulation and delivery), for making the SARS-CoV-2 vaccines possible. LNPs also can contribute adjuvant activity [41]. The various immune stimulating activities of mRNA have been well described, and include stimulation of TLR 3, 7, and 8, as well as RIG-I, MDA5, OAS and PKR [42,43,44]. Modifying the mRNA sequences also has been found to affect its immune reactivity [45]. An early mRNA clinical trial utilized mRNA as an adjuvant for a licensed rabies vaccine, with the intention of exploiting the TLR 7/8/RIG I agonist capabilities of the mRNA [46]. The use of modified nucleosides thus limited the inflammatory activity to the extent that the mRNA vaccine could be clinically developed for the COVID-19 vaccines, but the innate response has been assumed to play a key role in the development of the protection for the SARS-CoV-2 mRNA vaccines although the exact mechanisms have not been well delineated for different mRNA vaccines. Recent work utilizing a systems biology approach has demonstrated in humans that following the second injection of the BNT162b2 vaccine when neutralizing antibodies, CD4+ and CD8+ T cells were all seen, that there was a significant innate immune response greater than compared to similar times after the primary immunization [47]. Studies in knockout mice demonstrated that the mechanism of CD8+T cell activation was via MDA5 signaling, dependent on Type 1 IFN rather than TLR signaling or activation of inflammasomes [48].

DNA vaccines likewise stimulate innate immune responses, specifically TLR9, via the CpG motifs of these plasmids of bacterial origin. Supporting the rationale for their adjuvant activity in DNA vaccines is the fact that a CpG adjuvant is utilized in the recombinant protein-based hepatitis B vaccine, HEPLISAV-B, which is more efficacious compared to earlier recombinant hepatitis B vaccines and is thus used safely and effectively for people who do not respond to those previous vaccines [49]. DNA vaccines have been designed to have increased numbers of CpG motifs in efforts to increase their immunogenicity.

### 5.3. Antibody Responses from mRNA and DNA Vaccines

mRNA vaccines for SARS-CoV-2 generate protective antibodies, although the relatively rapid decline in circulating neutralizing antibodies has been a disappointment. Some of this rapid decrease may have been due to the immunization protocol with only 3–4 weeks between the initial and second dose. It was subsequently observed that a longer interval between the first and second dose of the Pfizer mRNA vaccine resulted in improved immune responses [50]. Similar results have been seen for many vaccines, including for an H5 influenza DNA vaccine, which resulted in higher hemagglutination inhibition antibody titers in humans when the interval was ≥12 weeks compared to ≤8 weeks [51]. The short interval was used for the mRNA COVID-19 vaccines because of the pandemic’s rapid spread. However, the short persistence of antigen in vivo from mRNA (compared to either DNA, or certainly live virus vaccines) may also mean that there was less time for the development of high affinity antibodies against the SARS-CoV-2 spike protein. Such high affinity antibodies generally arise as the amount of antigen becomes rate-limiting, thus allowing only those B cells with high affinity receptors to bind to the remaining antigen [52].

Many clinical trials for DNA vaccines have focused on using DNA as the means to stimulate T cells and as a prime for heterologous boosting by either a viral vector or protein for antibodies [53]. The potency for DNA to induce antibodies appears to be dependent on the particular protein, since human clinical trials for a West Nile Virus vaccine yielded titers even in elderly people, that were thought to be protective levels, similar to those obtained in horses for the licensed WNV DNA vaccine. A key issue for the antibody responses may have been the change in the promotor used for the WNV vaccine [54]. Three licensed veterinary vaccines generated antibodies of sufficient titers to consider the protective mechanism to be antibody-based, as shown in Table 5.

Despite the pandemic-changing results of the the mRNA vaccines for COVID-19, it is important to remember that for other mRNA prophylactic vaccine development efforts, the antibody responses were either not as stunning, or required more than one effort to design the vaccine, including the mRNA sequence, as was the case for the Moderna Zika sequential candidates. This does not mean that the technology will not ultimately work, but it provides an example of how as for any vaccine technology, one still needs to select the key antigens and then design the actual mRNA or DNA sequences, including for mRNA its formulation, for the optimal/necessary response.

### 5.4. T Cells: Induction by Nucleic Acid Vaccines and the Role for Immunotherapy

While the focus for prophylactic vaccines is often upon neutralizing antibodies, even those vaccines need to generate T cells of the helper phenotype to enable class switching and the development of high affinity antibodies. CD4+ T helper cells secrete cytokines needed for antibody production, and T follicular helper cells are specifically needed for the process of somatic hypermutation. CD8+ cytolytic T cells, by destroying tumor cells or virally infected cells, are important for limiting the growth of the tumor or the spread of virus (and in some cases they also cause some of the pathology of the disease) as noted above. Antibodies generally target antigens on the surface of a virus which are often variable, and mutations may enable virus progeny to then escape antibodies generated by a vaccine or against a previous version of the virus. T cells, rather than binding directly to viruses, instead bind to cells that have peptide fragments of the virus presented on their cell surface in conjunction with MHC molecules. As such, these peptide epitopes can be derived from any proteins, including internal proteins that are often more highly conserved because of their function. This means that T cell responses can be effective against more strains of a virus than many strain-limited antibodies. While T cells cannot provide so-called “sterilizing immunity” for preventing infection by a virus, their ability to provide cross-strain responses by targeting more highly conserved proteins, or regions of proteins that are more conserved, is important. This T cell-mediated cross-strain protection was indeed the initial in vivo pre-clinical demonstration of the ability of DNA vaccines to generate protective responses against a heterosubtypic strain of influenza (in addition to protective antibody responses against a homologous strain) [59]. mRNA vaccines provide strong protection against serious illness and death for vaccinated individuals infected with either homologous or variant strains of SARS-CoV-2 despite decreased neutralizing antibody against variant strains, again likely due largely or at least in part to the T cell responses.

mRNA vaccines have been shown to stimulate T follicular cells (Tfh), which as noted are important for the generation of antibodies [60]. While this capability likely played a role in the success for the COVID-19 vaccines, women receiving COVID-19 mRNA vaccines were counselled to delay having mammograms for 4–6 weeks after their second mRNA immunization because the unilateral swelling of axillary lymph nodes on the side of the immunization would pose a diagnostic difficulty. A more recent study shows that the time for resolution of the adenopathy is even longer, with different times for the two mRNA vaccines [61]. As mRNA vaccines become more widely utilized for either infectious diseases or particularly cancer, this clinical observation will need to be taken into account when evaluating either routine or diagnostic mammograms [61] or evaluating tumor spread in cancer patients [62].

## 6. DNA and mRNA Immunotherapeutic Vaccines under Development

### 6.1. Chronic Viral Infections

HIV DNA vaccines given to infected individuals were the first DNA vaccines to be tested in humans. Patients selected to have low or no immune responses to the HIV nef, rev, or tat, and those with low or no cellular immune responses against HIV regulatory proteins before immunization all responded to DNA immunization with memory responses; and 8 of the 9 patients made CTL, providing encouragement that DNA vaccines indeed could potentially induce immune responses even in patients who had been exposed chronically to viral antigens [63]. Modest antibody and cellular responses were noted in some HIV-infected patients following immunization with DNA encoding env and rev [64]. While encouraging for addressing the issue of trying to generate immune responses in the face of pre-existing exposure to antigen from the virus, the levels of the immune responses were not very impressive, although with the caveat that HIV is a virus that targets the CD4+ helper cells, so that the immune function in people infected with HIV may be one of the most challenging milieus for a therapeutic vaccine. Clinical trials for therapeutic HIV DNA vaccines have now largely focused on heterologous prime-boost regimens that include other vaccine modalities, such as viral vectors or recombinant proteins.

Targets for other chronic viral infections with DNA vaccines include hepatitis B and C. The current treatments for these diseases are anti-viral nucleos(t)ide analogs and interferon alfa-2b/ribavirin. While the latter’s mechanism of action is not fully understood, in addition to inhibiting viral replication, the interferon alfa-2b stimulates T cell immunity [65], a mechanism corresponding to the above-mentioned ability of T cell responses to occasionally spontaneously clear chronic hepatitis B infection. DNA vaccines were tested in several clinical trials for the treatment of chronic hepatitis B or chronic hepatitis C with electroporation (Table 6) because of their ability to stimulate T cells well, and certain trials thus also included interferon alfa2b. The results were generally disappointing, but the table is shown to illustrate key points about these trials: (1) various antigens were targeted, even surface antigen for hepatitis B, not just internal functional proteins, (2) administration was done with and without electroporation, (3) the immunizations were tested in some cases in conjunction with other interventions, whether anti-viral drugs or cytokines. Neither the phase 2 and 3 CMV vaccine studies for transplant recipients demonstrated therapeutic efficacy [66,67].

The major infectious disease targets for mRNA vaccines are for prophylactic indications, although Phase 1 and 2a trials performed in HIV-infected individuals were among the earliest mRNA vaccines to be tested clinically [68].

### 6.2. Cancer

Targets have included many different cancers for both DNA and mRNA. In some of the clinical trials, additional agents have been used such as checkpoint inhibitors, cytokines (including delivered also by a DNA plasmid), or adjuvants in an effort to further stimulate the immune responses beyond the provision of antigen and the immune stimulation of the nucleic acid vaccine itself. A number of DNA vaccines have targeted cervical intraepithelial neoplasia, the precursor to cervical carcinoma, caused in nearly all cases by previous infection with Human Papilloma virus. Many of these have used electroporation. Additional targets for DNA vaccines have included breast, prostate, colorectal, pancreatic cancers, and head and neck (HPV-mediated) cancers. Personalized DNA vaccines have been made in an effort to better match the tumor epitopes of the patient; as noted above, such a vaccine for B cell lymphoma was the earliest DNA cancer vaccine to enter the clinic [37].

Many RNA vaccines have been made using the RNA as means to transfect Dendritic cells ex vivo, which then are given to the patient to induce immune responses, as mentioned above. However, to avoid the obvious logistical and time delay disadvantages of such an approach, human trials began focusing on direct in vivo administration of the mRNA. One of the earliest clinical tests of direct injection of mRNA was based on the premise of using the unmodified mRNA as both the vector to deliver the prostate tumor antigens and as the adjuvant (via the TLR7 stimulation, and possibly other mechanisms) for the immune responses [69]. The ability of mRNA to be easily made for various antigens has led to both targeting multiple antigens in addition to making personalized vaccines either exactly specifically for the patient or by using a mix of antigens from a pre-made library of relevant tumor antigens. mRNA vaccines have thus targeted many different types of cancers targeting both tumor-associated and tumor-specific antigens. The dozens of studies can be found at clinicaltrials.gov and useful summary tables based on differences in formulation and phase of study can be found in a recent review [70]. mRNA encoding tumor antigens are also being tested in combination with other means to drive immune responses, such as combining the mRNA with other immune modalities such as checkpoint inhibitors.

## 7. An Additional Potential Immunotherapeutic Mechanism and Role for Nucleic Acid Vaccines?

Clinical data going back to the smallpox vaccine and then continuing with observations of children immunized with BCG have reported that immunized individuals were less susceptible to or had improved mortality from other infectious diseases [71]. The observations have been based mainly on live vaccines, and during the COVID-19 era even resulted in efforts to use live vaccines such as polio [72] and BCG [73] to protect against COVID-19. In addition, it was found that people who had been previously immunized with BCG had a lower seroprevalence for SARS-CoV-2 [74].

This has led to increased interest in the concept of improving immune fitness [75]. Immune fitness may have impacts upon an individual’s ability to clear chronic viral infections or to fight cancer, beyond the specific antigen being targeted by the DNA or mRNA vaccines. Because DNA and mRNA vaccines in some ways are more like a live vaccine (for example in their induction of Th1 vs. Th2 response compared to for example alum-adjuvanted non-live vaccines), it would be interesting to determine whether they also improve immune fitness. Further systems biological studies of immune cells following DNA and mRNA priming and boosting as prophylactic and therapeutic vaccines, to compare the transcriptional signatures may yield insights into this potential capability.

## 8. Conclusions: What Can We Expect in the Future for Immunotherapeutic Nucleic Acid Vaccines for Chronic Infections and Cancer?

The impressive efficacy and rapid development timelines for the SARS-CoV-2 mRNA vaccines have raised the hopes for using nucleic acid vaccines for additional prophylactic vaccines as well as for therapeutic applications. Meanwhile, the authorization of the COVID-19 DNA vaccine following the licensures of various veterinary DNA vaccines provides hope that this technology will likewise be useful for additional prophylactic and therapeutic human and veterinary vaccines. The ability to rapidly make constructs combined with their ability to stimulate innate immune responses on top of delivering the gene encoding the antigen, plus the generic nature and ease of manufacture, highlight key attractive features. However, the modest immune responses seen for certain targets, both prophylactic and therapeutic with both technologies, along with the many immunological challenges for therapeutic vaccines related to immune escape and suppression mean that many issues may need to still be addressed. It is crucial that the immune dysfunction found in states of chronic viral infection and cancer be treated by the immunotherapeutic vaccine, in addition to provision of antigen [76]. It may be necessary to combine nucleic acid vaccines with other vaccine modalities (i.e., prime-boost strategies) or components with additional immune-modulating activities such as adjuvants or check-point inhibitors to successfully treat chronic viral infections and cancer. Further exploration of the quality and versatility of the specificity of antibody needs to be done to determine whether boosts, or re-use of the vaccines will be able to generate antibodies that are more specific against mutated viral or tumor surface antigens should the need arise. Both technologies are excellent inducers of T cell immune responses, including with the Th1 phenotype of T cell help, which are likely key for such therapies. It will additionally be of interest to explore whether nucleic acid vaccines can augment immune fitness more generally, perhaps via their innate immune stimulation to improve more broadly immune responses and the immune dysfunction seen with aging.

## Figures and Tables

**Table 1:** Examples of Pathogen Etiologies of Infectious Diseases and Malignancies.

Pathogen	Infectious Disease	Malignancy
Hepatitis B Virus	Acute and Chronic Hepatitis	Hepatocellular Carcinoma
Hepatitis C Virus	Acute and Chronic Hepatitis	Hepatocellular Carcinoma
Human Papilloma Virus (outcome is strain-dependent)	Warts	Cervical Carcinoma Non-Melanoma Skin Cancer (NMSC), Anogenital Carcinoma
Epstein–Barr Virus	Mononucleosis	B-cell Lymphoproliferative Diseases including various B Cell Lymphomas, Post-transplantation Lymphoproliferative Disease, Nasopharyngeal Carcinoma
*H. pylori*	Peptic Ulcers	Gastric Carcinoma, Gastric Mucosa-Associated Lymphoid Tissue (MALT) lymphoma.

**Table 2:** Rationale for the Potential Success of Immunotherapy of Chronic Viral Infections and Cancer.

The observation of cancers arising in immunosuppressed states, thus demonstrating that normal immune responses play a role in inhibiting the development of cancer ○ HIV infection leading to AIDS-defining cancers ○ Cancers arising in patients on immunosuppression following organ transplantation
The success of various immune activation approaches ○ Non-specific immune stimulation ▪ Coley’s toxin ▪ Bacillus Calmette–Guérin (BCG) instillation in the bladder to prevent progression and recurrence of bladder cancer ○ Cytokines ▪ Interferon alpha (IFNα) for chronic Hepatitis B and C ▪ Interleukin-2 (IL-2) for metastatic renal cell cancer (via T cells) ▪ IL-2 for melanoma (via T cells) ○ Other T cells approaches for cancer ▪ Checkpoint inhibitors

**Table 3:** Existing Antigen-Specific Immunotherapy Modalities.

Monoclonal Antibody Treatments for Viral Infections and Cancer
Bi-specific antibody cancer therapies
CAR-T cells ○ Lymphoma ○ Leukemia ○ Multiple myeloma

**Table 4:** DNA and mRNA Authorizations/Licensures: Prophylactic and Therapeutic.

DNA	mRNA
COVID-19 authorized, needle-free delivery (India)	COVID-19 Vaccines: Licensed: multiple countries
Equine West Nile Virus (2005, USA)	
Fish Hematopoietic Necrosis Virus (2005, Canada)	
Fish Salmon Alphavirus Subtype 3 (2016, Europe)	
Dog Melanoma: Cancer immunotherapeutic vaccine (2007, USA)	
Non-vaccine: Pig Growth Hormone Releasing Hormone (2008, Australia; Pregnant sows; electroporation)	

**Table 5:** Immune Mechanisms of Licensed Veterinary DNA Vaccines.

Vaccine	Mechanism/Comments
Fish Hematopoietic Necrosis Virus	Neutralizing antibody Complete or near-complete prevention of viral spread to unimmunized co-habiting fish [55,56]
Fish Salmon Alphavirus Subtype 3	Neutralizing antibody Better protection than traditional vaccine; Decreased viral spread to unimmunized fish [57]
Equine West Nile Virus	Neutralizing antibody [58] (Antibodies in human trials for WNV with a DNA vaccine including the eldery yielded antibody titers also expected to be protective. Promoter for DNA vaccine had been optimized [54])

**Table 6:** Clinical trials for DNA vaccines for chronic viral infections.

Virus	Name	Title	Antigen(s)	Formulation/Additional Interventions	NCT Number(s)	Phase	Start/Finish (or Estimated Study Completion)	Status
Hepatitis B	pCMV-S2.S DNA	A Randomized Controlled Trial of Dual-plasmid HBV DNA Vaccine Mediated by in Vivo Electroporation in Chronic Hepatitis B Patients Under Lamivudine Chemotherapy	Small (S), Middle (preS2 + S) Envelope proteins	Enrolled following viral breakthrough while on lamivudine	NCT00988767	1	February 2001–October 2004	Completed
Hepatitis B	JNJ-64300535	A First-In-Human Study to Evaluate Safety, Tolerability, Reactogenicity, and Immunogenicity of JNJ-64300535, a DNA Vaccines, Administered by Electroporation-Mediated Intramuscular Injection, in Participants with Chronic Hepatitis B Who Are on Stable Nucleos(t)ide Therapy and Virologically Suppressed	Core, Polymerase (Pol)	Electroporation, nucleos(t)ide therapy	NCT03463369	I	18 April 2018–23 March 2021	Completed
Hepatitis B	JNJ-64300535	A Phase 1b, Open-label, Single-arm, Multicenter Study to Assess Efficacy, Safety, and Tolerability of Treatment With JNJ-73763989, JNJ-64300535, and Nucleos(t)Ide Analogs in Virologically Suppressed, HBeAg-negative Participants With Chronic Hepatitis B Virus Infection	Core, Polymerase (Pol)	Electroporation, nucleos(t)ide analogs, experimental RNAi	NCT05123599	Ib	6 December 2021–2 August 2024	Recruiting
Hepatitis B	INO-1800	Phase I, Randomized, Open-Label, Active-Controlled, Dose Escalation Study to Evaluate the Safety, Tolerability & Immunogenicity of INO-1800 Alone or in Combination With INO-9112 Delivered IM Followed by EP in Select Nucleos(t)Ide Analogue-Treated, Chronic Hepatitis B Patients	Surface Ag, Core	Electroporation, nucleos(t)ide Analogs, +/−IL-2 delivered as DNA (INO-9112)	NCT02431312	I	12 January 2015–22 May 2018	Completed
Hepatitis C	INO-8000	Phase I Trial of a Therapeutic DNA Vaccine for Chronic Hepatitis C Virus (HCV) Infection	Nonstructural proteins 3 (NS3), 4A (NS4A), 4B (NS4B) and 5A (NS5A)	Electroporation, INO-9012 (IL-12 adjuvant DNA)	NCT02772003	1	6 June 2016–4 March 2020	Active, Not Recruiting
Hepatitis C	CHRONVAC-C^®^	A Phase I/IIa Open-Label, Dose Ranging, Parallel, Safety, Tolerability and Efficacy Study of i.m. Administered CHRONVAC-C^®^ in Combination With Electroporation in Chronic HCV Genotype 1 Infected and Treatment Naïve Patients With Low Viral Load	Nonstructural proteins NS3/NS4a	Electroporation, Peg-IFNα/Ribavirin	NCT00563173	1/2a	October 2007–April 2010	Unknown
Hepatitis C	CHRONVAC-C^®^	A Phase II Open-Label, Randomized, Parallel Group, Safety, Tolerability and Efficacy Study of i.m. Administered CHRONVAC-C in Combination With Electroporation Followed by Standard of Care in Chronic Hepatitis C Virus Genotype 1 Infected and Treatment Naïve Subjects	Nonstructural proteins NS3/NS4a	Electroporation, Peg-IFN-α-2a/Ribavirin	NCT03463369	2	April 2011–June 2012	Unknown
CMV	ASP0113	A Phase 1, Single-Blind, Parallel-Group, Pharmacokinetic and Immunogenicity Study With ASP0113 in CMV-Seropositive and CMV-Seronegative Healthy Subjects and CMV-Seronegative Dialysis Patients	Two plasmids: Glycoprotein B and phosphoprotein 65 (pp65)	Formulated with CRL1005 poloxamer and benzalkonium chloride; Patients seropositive and seronegative	NCT02103426	1	30 December 2013–10 May 2016	Completed
CMV	ASP0113	A Randomized, Double-Blind, Placebo-Controlled, Phase 2 Trial to Evaluate the Efficacy and Safety of a Vaccine, ASP0113, in Cytomegalovirus (CMV)-Seronegative Kidney Transplant Recipients Receiving an Organ From a CMV-Seropositive Donor	Two plasmids: Glycoprotein B and phosphoprotein 65 (pp65)	Formulated with CRL1005 poloxamer and benzalkonium chloride. Immunization started after transplant performed.	NCT01974206	2	20 November 2013–5 November 2020	Completed; No efficacy
CMV	ASP0113	A Randomized, Double-Blind, Placebo-Controlled, Phase 3 Trial to Evaluate the Protective Efficacy and Safety of a Therapeutic Vaccine, ASP0113, in Cytomegalovirus (CMV)-Seropositive Recipients Undergoing Allogeneic, Hematopoietic Cell Transplant (HCT)	Two plasmids: Glycoprotein B and phosphoprotein 65 (pp65);	Formulated with CRL1005 poloxamer and benzalkonium chloride; Patients seropositive but no active infection or disease	NCT01877655	3	11 September 2013–1 March 2022	Completed; No efficacy
